# Diseases of the Arteries, Including Angina Pectoris

**Published:** 1916-12

**Authors:** 


### Reviews of Books.

**Diseases of the Arteries, including Angina Pectoris.** By Sir CLIFFORD ALLBUTT, K.C.B., F.R.S., F.R.C.P., D.Sc., etc. In two volumes. Pp. xi. 534, vi. 559. London: Macmillan & Co. 1915. Price 30s.

Surely no one has previously written two such volumes on the medical aspects only of the arterial system. The undertaking of such a task implies a degree of knowledge and erudition which few men would claim to possess. That it has been done by the Regius Professor of Physic in the University of Cambridge would at once commend the work as a masterly one; but, further, the fact that the Regius Professor is Clifford Allbutt, whose name has for so many years connoted the highest possible excellence in any work he undertakes, places these volumes on a pedestal of their own. We venture to think that no living man was better qualified to undertake the writing of an authoritative monograph on this difficult and little-known subject, and yet the author commences his preface by commenting on "these faults of a book which lie in the quality of its author." He says: "Of course, we cannot know all, nor anything like all; much of the detailed structure and of the fuller outlines of every subject is still obscure or out of sight; but can the author reasonably pretend that he possessed interpretation enough ro enable him to deal with the facts provisionally?" He further points out that "by leaving some data in the background and bringing others into high relief, by painting a little colour here and throwing a little shadow there, it is not difficult to construct an argument far more taking, far more 'readable,' than pages of outlines still vague, of meanings still tentative, of facts still insecure and unbalanced. Immature 'scientific' truth, like the ugly duckling, is plain in feature, in form unattractive, and very cold in colour."

The subject "Arteriosclerosis" occupies the whole of vol. i. and the first eighty pages of vol. ii. Then follow some seventy pages on "Aortitis," and nine chapters on "Angina Pectoris" complete the author's scheme.

Much of the work centres round the condition described and named by the author Arterial Plethora or Hyperpiesis, the malady being called Hyperpiesia; this name is to connote, not the high arterial pressure only, but the whole morbid series, not chronic renal disease, of which high arterial pressure is one term. This condition persisting, we get one variety of arteriosclerosis, the effect of persistently high blood-pressures. Here we come upon a condition which appears to have been vastly on the increase in recent years, and more especially in

America, and of course there is much room for doubt as to how much of what is reported as such is a convenient clinical term not necessarily founded on pathological investigation or knowledge. Some accuracy has been given to the question by the introduction of various mechanical means of registering the phases of arterial pressures; the sphygmograph gave much useful knowledge, but the various forms of pressure recorders, of which Oliver's sphygmomanometer is the type, have done much more to give accuracy than the tactus eruditus can ever do.

This pressure-gauge is now in general use, and nowhere more than in the United States, where the question is frequently asked by the Insurance Societies, "What is the blood pressure?" The numerous instruments advertised, and the books written thereon, are an index that the profession in general realises the value of mathematical accuracy where possible, and that the use of these instruments is no longer left to the physiologist and pathologist, but comes into the usual routine of clinical work. The author has now for many years urged that the profession paid too little attention to the question of blood-pressure, but it is obvious that in the future it will form one of the most important grounds on which the prognosis in brain, kidney, and arterial diseases will be estimated.

In vol. ii., chap. ix., the author endeavours to differentiate between the hyperpietic, the toxic, and the senile or decrescent sclerosis; but he adds that "so long as arteriosclerosis is accepted as a disease statistics are impossible: the Insurance tables are compiled on heterogeneous materials; or, if blood-pressures are taken, only the systolic are recorded."

The author has in many ways and various times pronounced his views on angina pectoris. They have occupied many addresses, and have been partially published, but are now republished in full, after revision, and form the major part of the second volume.

